# Solitary preperitoneal neurofibroma: a case report

**DOI:** 10.1186/s13104-015-1098-8

**Published:** 2015-04-01

**Authors:** Noureddine Njoumi, Mohamed Elabsi, Gilles Attolou, Hafsa Elouazzani, Faricha Hassan Elalami, Mohamed Rachid Chkoff

**Affiliations:** Department of Visceral Surgical Emergency, Ibn Sina Hospital, Rabat, Morocco; Department of Anatomical Pathology, Ibn Sina Hospital, Rabat, Morocco

**Keywords:** Solitary neurofibroma, Benign tumor, Preperitoneal space

## Abstract

**Background:**

Neurofibroma is a rare benign tumor. The isolated presence of such lesions in the preperitoneal space with no evidence of systemic disease has never been reported in the literature.

**Case presentation:**

A 29-year-old white man presented with a 12 months history of progressive abdominal distension. Clinical examination revealed a bulky hypogastric mass. Abdominal computed tomography and pelvic magnetic resonance imaging showed a large, well defined preperitoneal tumor measuring 18 x 17cm extending in the pelvis. A computed tomography guided biopsy was performed which revealed a neurofibroma. Exploratory laparotomy showed a well encapsulated elastic soft tumor in the preperitoneal space which measured 17 x 18cm and weighted 2 Kg. The tumor was completely excised. No recurrence occurred after one year of follow-up.

**Conclusion:**

Solitary preperitoneal neurofibroma is an extremely rare benign tumor. Its clinical and radiological signs are nonspecific. Preoperative histology can be useful to guide the surgical approach which is the only curative treatment.

## Background

Neurofibroma is an uncommon benign tumor arising from the peripheral nerve sheaths. The isolated presence of neurofibromatous lesions in the preperitoneal space (between the parietal peritoneum and transversalis fascia), without any other sign of von Recklinghausen’s disease has rarely been reported [1]. Authors report an unusual case with a review of literature.

## Case presentation

A 29-year-old white man, with no history of neurofibromatosis or other systemic disease, was complaining of insidious and progressive abdominal distension which appeared 12 months prior to admission. He had hypogastric abdominal pain and pollakiuries in the preceding five months. There were no vomiting or transit disorders, and no neurological symptoms.

On clinical examination, the patient was in good general condition with a normal temperature, a body mass index of 24 and no café au-lait spots or dermal neurofibromas on his body. The palpation of the abdomen revealed a well-defined, fixed and painless hypogastric mass, 20 cm in diameter. Digital rectal examination was normal.

All the standard hematological and serum biochemical data were normal.

**Figure 1:**
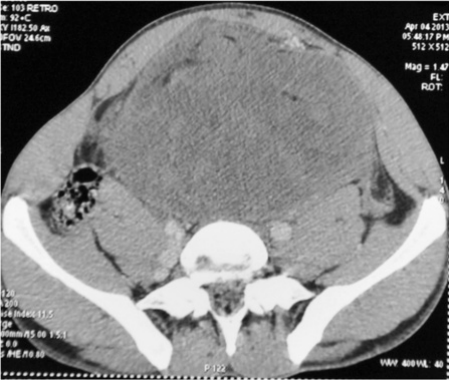
**Abdomino-pelvic computed tomography showed a massive, inhomogeneous, hypodense abdominal tumor.**

An abdomino-pelvic computed tomography (CT) scan (Figure 1) revealed a massive, inhomogeneous, hypodense, preperitoneal tumor, measuring 17 × 18cm extending in the pelvis, crushing both ureters and compressing the bladder. This mass was moderately enhanced with contrast. There were no signs of pelvic invasion or intraperitoneal effusion. Both kidneys were increased in size with dilation of renal cavities but the renal cortex was preserved.

**Figure 2:**
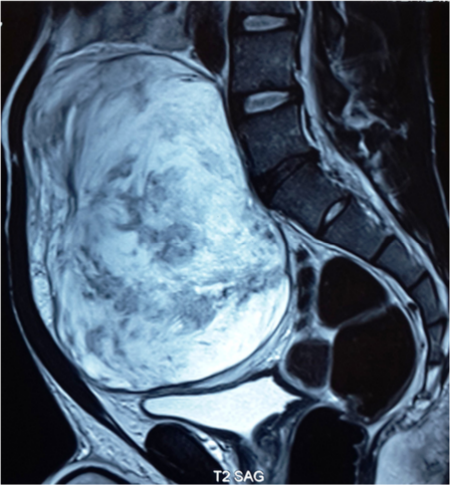
**Pelvic magnetic resonance imaging showing a large, well encapsulated preperitoneal tumor.**

Pelvic magnetic resonance imaging (Figure 2) revealed a large, well encapsulated preperitoneal tumor, slightly compressing the bladder and the pelvic ureters without invasion of the adjacent structures and without pelvic bone localization.

A CT-guided biopsy was performed revealing a conjunctive tissue separated by a fuso-cellular proliferation compound of Schwann cells with regular nuclei immersed in a richly vascularized and myxoid fibrillar background. There was no evidence of malignancy within the tissue obtained. Immunohistochemically, the tumor cells were strongly positive for the S100 and the neurofilament proteins evoking a neurofibroma.

**Figure 3:**
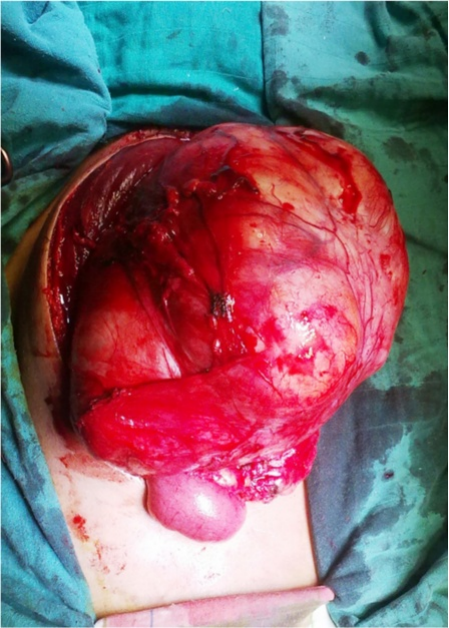
**Intraoperative view showing the well encapsulated elastic soft tumor in the preperitoneal space.**

Surgical resection was decided by the multidisciplinary meeting for cancer care. Exploratory laparotomy revealed a well encapsulated elastic soft tumor in the preperitoneal space. Upon incision of the linea alba, there was a protrusion of the mass. It was easily cleaved before the opening of the anterior parietal peritoneum. At the end of excision, there was a peritoneal break due to size of the mass (Figure 3).

**Figure 4:**
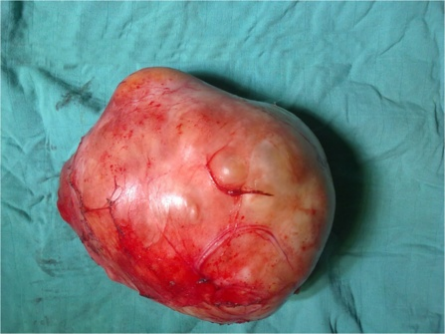
**Macroscopic appearance of the preperitoneal neurofibroma; size = 17x18cm, weight = 2 kg.**

The tumor measured 17 × 18 cm and weighted 2 Kg (Figure 4). It was completely excised without macroscopic capsule breaking. No lymph nodes or other palpable intra-abdominal masses were discovered.

**Figure 5:**
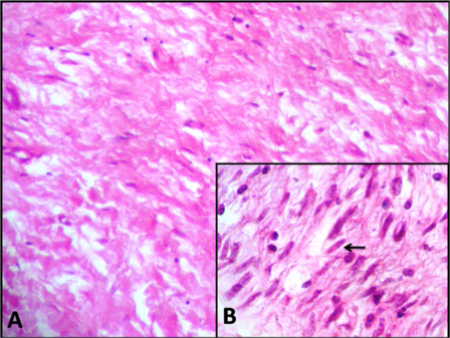
**Benign tumor cells immersed in a myxoid collagen background (A) with elongated and wavy nuclei (B).**

The histological and immuno-histochemical analysis of the surgical specimen confirmed the preoperative diagnosis (Figure 5). Thus, the tumor was diagnosed as a solitary neurofibroma. The post-operative course was uneventful. No adjuvant treatment was indicated. The patient remains well without any sign of tumor recurrence on a CT scan performed 12 months after surgery.

### Comments

Neurofibroma is a tumor of neural origin derived from the cells that constitute the nerve sheath. It’s an uncommon benign tumor found as a solitary tumor or as a partial manifestation of neurofibromatosis type I (NF1, also called von Recklinghausen’s disease) [2]. The cause of solitary neurofibroma is still unknown [3], it is defined by Marocchio et al. [4] as a hyperplastic hamartomatous malformation rather than neoplastic. NF1 is an autosomal dominant genetic syndrome caused by mutations in genes coding for neurofibromin [5], it is characterized by cutaneous manifestations as cafe-au-lait spots with a large number of nervous system tumors [6]. The hereditary factors and systemic symptoms present in the disseminated neurofibromas are absent in the solitary type [7].

This kind of neoplasms is known to mainly affect the cutaneous nerves of the trunk, neck, and head [8,9]. Rare cases in the deep organs or in the peritoneal cavity were also reported [10]. Concerning the retroperitoneal location, exceptional cases of benign nerve sheaths tumors were reported in large series of primitive retroperitoneal tumors [11,12]. To our knowledge, the presence of solitary neurofibroma in the preperitoneal space has never been reported in the literature.

Neurofibroma appears to affect adolescents and young adults without a gender preference. It grows along the peripheral nerves as a non-encapsulated focal mass with well-defined margins [8,9].

The clinical manifestations of solitary neurofibromas are not specific and change according to their locations, their effect on gastrointestinal motility and their possible impingement on contiguous structures, resulting in palpable masses, abdominal pain, and transit disorders due to extra-luminal pressure [13-15]. The deep seated neurofibroma on peripheral nerves and spinal roots frequently leads to neurological disability [16].

Intra pelvic tumors are often diagnosed at a late stage which is source of surgical resection difficulties [17].

Preoperative imaging is usually insufficient to establish the diagnosis with certainty, only the histology can do. In fact, the preoperative histological evidence can influence the treatment modifying the surgical approach, conservative versus aggressive [10].

Surgical removal is the only treatment option. Unlike our case, neurofibromas are difficult to manage surgically as they are extensively infiltrative and highly vascular [15,18].

Some authors posit that surgical resection is indicated only when the tumor causes pain, neurological deficiencies or when there is a strong suspicion of malignancy [19].

Solitary neurofibromas have a good prognosis, with rare instances of haemorrhage, malignant changes and local recurrences after excision [18,20].

## Conclusion

The solitary preperitoneal neurofibroma is an extremely rare benign tumor arising from nerve sheaths. Its clinical and radiological signs are not specific. Preoperative histological diagnosis can be useful to help choosing the surgical approach, which is the gold-standard treatment option. Unlike other sites, tumor excision in the preperitoneal location remains without difficulties.

## Consent

Written informed consent was obtained from the patient for publication of this Case report and any accompanying images. A copy of the written consent is available for review by the Editor-in-Chief of this journal.

## Abbreviations

CT: Computed tomography
NF1: Neurofibromatosis type I
